# A Machine Learning Model for Predicting Posthepatectomy Liver Failure After Hepatectomy With Extrahepatic Bile Duct Resection for Perihilar Cholangiocarcinoma: With and Without Indocyanine Green

**DOI:** 10.1002/ags3.70187

**Published:** 2026-01-30

**Authors:** Yuki Homma, Hiroki Ohya, Ryusei Matsuyama, Isamu Hosokawa, Tsukasa Takayashiki, Yusuke Ome, Shuichiro Uemura, Ryota Higuchi, Goro Honda, Eiryo Kawakami, Masayuki Ohtsuka, Itaru Endo

**Affiliations:** ^1^ Department of Gastroenterological Surgery Yokohama City University Graduate School of Medicine Yokohama Kanagawa Japan; ^2^ Department of General Surgery, Graduate School of Medicine Chiba University Chiba City Chiba Japan; ^3^ Department of Surgery, Institute of Gastroenterology Tokyo Women's Medical University Shinjuku‐ku Tokyo Japan; ^4^ Division of Gastroenterological Surgery Tokyo Women's Medical University Yachiyo Medical Center Yachiyo Chiba Japan; ^5^ Department of Artificial Intelligence Medicine, Graduate School of Medicine Chiba University Chiba City Chiba Japan

**Keywords:** machine learning, perihilar cholangiocarcinoma, posthepatectomy liver failure

## Abstract

**Aim:**

Posthepatectomy liver failure (PHLF) is a serious complication of major hepatectomy and is closely related to perioperative mortality. Whether or not indocyanine green (ICG) is used to measure liver function varies by country. Using machine learning, we aimed to develop two highly accurate, user‐friendly models for predicting PHLF depending on whether ICG data were used.

**Methods:**

This was a retrospective, three‐center study. SHapley Additive exPlanations (SHAP) evaluated the feature importance of Random Forest (RF) analysis. SHAP quantitatively assessed the impact of each feature on model predictions and identified the three most important features in both ICG‐used and ICG‐unused models. A Decision Tree (DT) model was constructed using two of these three key features to enhance clinical interpretability. PHLF was defined as Grade B or C according to the International Study Group of Liver Surgery.

**Results:**

Feature importance was calculated using the SHAP analysis, in the ICG‐used model the ICG clearance rate (ICGK) multiplied by the percentage of remaining liver measured by CT volumetry (ICGK‐F), CRP, and operative procedure were identified as the three highest factors, whereas the predicted liver resection rate, CRP, and total bilirubin were identified as the most important features in the ICG‐unused model. In the constructed DT model, we categorized the cases into negative and positive, and the negative cases were further classified into three categories based on their risks.

**Conclusion:**

These models may offer a simple and practical approach for predicting the risk of PHLF and hold promise for future clinical application.

## Graphical abstract

Using machine learning‐based decision tree models, patients with perihilar cholangiocarcinoma undergoing major hepatectomy with extrahepatic bile duct resection were stratified according to the risk of posthepatectomy liver failure. Separate models were developed with and without indocyanine green data, enabling clinically interpretable preoperative risk assessment.

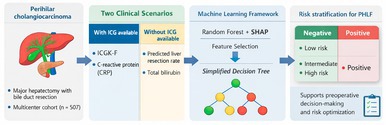

## 1 Introduction

Posthepatectomy liver failure (PHLF) is one of the most serious complications of hepatic resection and is closely associated with perioperative mortality [1]. Especially surgical treatment for hilar cholangiocarcinoma is associated with a high risk for PHLF [2].

Although advances in operative procedure and perioperative management have reduced the mortality rate to 5%–7% in Japanese hospitals with high caseload rates, PHLF remains a life‐threatening complication [3, 4].

PHLF is related to hepatic functional reserve and should be evaluated in terms of both liver function and future liver volume (FLV) [5, 6]. To assess the functional reserve of the remaining liver, the Japanese clinical practice guidelines for biliary tract cancer (4th edition, in Japanese) recommend using the indocyanine green plasma clearance rate (ICGK) multiplied by the percentage of remaining liver measured by CT volumetry (ICGK‐F), which is known as the indocyanine green clearance of the future liver remnant [7]. It is recommended that the clearance rate be used; the cutoff value for ICGK‐F is set at 0.05 to reduce the risk of PHLF and death after hepatic resection for cholangiocarcinoma [8], but ICGK‐F alone is not a risky way to assess functional reserve. Therefore, a multi‐layered evaluation method using multiple criteria is required. Furthermore, ICGK‐F alone did not predict PHLF with sufficient accuracy [5, 8]. Therefore, complementary factors should be considered in addition to ICGK‐F to predict PHLF more precisely and determine appropriate surgical indications. The remnant liver proportion and indocyanine green (ICG) tests are commonly used in Japan because of their ease of use and cost‐effectiveness, whereas the ICG test is rarely used in Western countries [9]. In Western countries, scintigraphy is generally preferred over ICG for evaluating liver function [10]. There have been several reports on factors predicting PHLF without ICG. The use of FLV/body surface area and FLV/body weight has been reported in perihilar cholangiocarcinoma and hepatocellular carcinoma [11, 12].

Machine learning (ML) is a data analysis technique in which a computer inputs large amounts of historical data (features), learns hidden patterns, and develops rules to identify unknown data. Therefore, ML may outperform traditional risk stratification tools by integrating different algorithms, such as decision tree (DT), artificial neural network (NN), and random forest (RF) analysis. Recent reports have also been published on predictive models for PHLFs using ML [13]. However, these reports were not based on surgery for perihilar cholangiocarcinoma, and the generality of the prediction model was lacking.

In this study, we aimed to develop two highly accurate and user‐friendly models for predicting PHLF depending on whether ICG was used (ICG‐ and ICG‐unused models) in perihilar cholangiocarcinoma.

## 2 Method

### 2.1 Patient Selection

This was a retrospective, three‐center observational study. Clinical records of patients with perihilar cholangiocarcinoma who underwent surgery at Tokyo Women's Medical University Hospital, Chiba University Hospital, and Yokohama City University Hospitals were examined retrospectively.

The eligibility criteria were as follows: (1) patients aged ≥ 18 years, (2) patients with preoperatively diagnosed perihilar cholangiocarcinoma, and (3) patients who underwent major hepatectomy, including caudate lobectomy with bile duct resection. Patients who underwent hepatectomy combined with pancreatoduodenetomy were excluded. The collected data were retrospectively examined in accordance with investigational protocols approved by the Institutional Review Board and Ethics Committee of Yokohama City University (approval number F220100002). This study was conducted in compliance with the ethical standards outlined in the Declaration of Helsinki. Informed consent was obtained based on the opt‐out principle.

### 2.2 Definition of PHLF

PHLF was defined according to the International Study Group for Liver Surgery (ISGLS) [1]. This study considered clinically relevant PHLF. Grades B and C were defined as PHLF.

### 2.3 Learning Features and Interpretation of a Novel Prediction Model

To develop a model for predicting the presence of PHLF, only clinicopathological data collected from preoperative examinations were used as the learning features. To predict postoperative liver failure, the following preoperative data were used to construct the two models based on whether ICG was used.

#### 2.3.1 ICG‐Used Model

Age; body mass index (BMI); hemoglobin (Hb), platelet count, albumin, total bilirubin, Alanine aminotransferase (ALT), C‐reactive protein (CRP) and creatinine levels; preoperative treatment; preoperative biliary drainage; preoperative cholangitis; preoperative chemotherapy; ICGK‐F; operative procedure; and facility. Preoperative laboratory data, ICG test, and CT volumetry were obtained as part of the routine preoperative assessment. The exact dates of ICG test and CT were not collected as structured variables in this retrospective study. However, in routine clinical practice at the participating institutions, ICG testing is typically performed within approximately 1 month before surgery and is reexamined after portal vein embolization when applicable.

#### 2.3.2 ICG‐Unused Model

Age; BMI; Hb, platelet count, albumin, total bilirubin, ALT, CRP and creatinine levels; preoperative treatment; preoperative biliary drainage; preoperative cholangitis; preoperative chemotherapy; predicted resection rate; operative procedure; and facility. Preoperative laboratory data and CT volumetry were obtained from the most recent examinations performed before surgery and were used for model development.

### 2.4 Model Building and Evaluation

In this study, three models were built using Python, and their performances were compared using logistic regression (LR), Neural Network (NN), and Random Forest (RF) analyses. The area under the receiver operating characteristic curve (AUC) and accuracy (ACC) were used to evaluate and compare the performance of each model. To evaluate the robustness of the prediction models, internal validation was performed using 10‐fold cross‐validation. Model performance was assessed by calculating AUC, accuracy, PPV, and NPV across the validation folds.

### 2.5 Evaluation of Feature Importance and Construction of the Decision Tree Model

SHapley Additive exPlanations (SHAP) was used to evaluate the feature importance of the RF models. Using SHAP, the impact of each feature on the model predictions was quantitatively assessed, and the two factors contributing to the three most important features were identified in both the ICG‐used and ICG‐unused models. A Decision Tree (DT) model was constructed using the rpart package in R. The maximum depth of the decision tree was set to 3 (max depth = 3), and variables and thresholds that maximized entropy reduction were selected (split = “information”).

It was then evaluated using metrics such as AUC and ACC to assess its performance compared to that of other models.

## 3 Results

This study included 601 patients with perihilar cholangiocarcinoma, who underwent right or left hemihepatectomy or trisectionectomy between January 2001 and August 2021. After excluding patients with unmeasured preoperative data, 507 were included in the analysis. The baseline characteristics of the PHLF and non‐PHLF groups are presented in Table 1. PHLF occurred in 156 of the 507 patients (31%). Significant differences in sex; Hb, CRP, albumin, and total bilirubin, preoperative drainage; portal vein embolization (PVE), operative procedures, facilities, ICGK‐F, and expected liver resection rates were noted between the two groups. Thirty‐day and 90‐day mortalities were significantly higher in the PHLF group than in the non‐PHLF group (Table 1). PVE was performed in a substantial proportion of patients, particularly in those undergoing right‐sided or extended hepatectomy. The frequency of PVE was markedly higher in right hemihepatectomy (173/245, 71%), right trisectionectomy (16/20, 80%), and left trisectionectomy (52/64, 81%) than in left hemihepatectomy (10/167, 6%) and central bisegmentectomy (3/11, 27%), reflecting procedure‐specific surgical strategies.

**TABLE 1:** The baseline characteristics of the PHLF and non‐PHLF groups.

	Non‐PHLF group (*n* = 351)	PHLF group (*n* = 156)	*p*
Sex, man/woman	228/123	118/38	0.017
BMI (kg/m^2^) (mean ± SD)	21.8 ± 2.9	21.9 ± 3.3	0.683
Hb (g/dL) (mean ± SD)	12.1 ± 1.6	11.7 ± 1.8	0.05
Platelet count (×1000/μL) (mean ± SD)	250 ± 135	252 ± 80	0.128
CRP (mg/dL) (mean ± SD)	0.79 ± 1.27	1.24 ± 1.82	< 0.001
Albumin (g/dL) (mean ± SD)	3.74 ± 0.45	3.45 ± 0.47	< 0.001
ALT (U/L) (mean ± SD)	56.3 ± 94.1	54.0 ± 49.9	0.6
Creatinine (mg/dL) (mean ± SD)	0.72 ± 0.19	0.76 ± 0.44	0.647
Total bilirubin (mg/dL) (mean ± SD)	0.99 ± 0.69	1.36 ± 1.16	< 0.001
ICGK‐F (ICGK×FLV) (mean ± SD)	0.086 ± 0.031	0.067 ± 0.023	< 0.001
Expected liver resection rate (%) (mean ± SD)	43.7 ± 14.9	52.1 ± 11.7	< 0.001
Preoperative chemotherapy	80 (22.8%)	25 (16.3%)	0.082
Preoperative biliary drainage	273 (77.7%)	144 (92.3%)	< 0.001
Preoperative cholangitis	93 (26.4%)	46 (29.4%)	0.557
Portal vein embolization	165 (47%)	89 (57%)	0.0369
Operative procedure			
Right hemihepatectomy	153	92	< 0.001
Left hemihepatectomy	142	25	
Right trisectionectomy	12	8	
Left trisectionectomy	36	28	
Central bisectionectomy	8	3	
30‐day Mortality (%)	2 (0.6%)	8 (5.1%)	< 0.001
90‐day Mortality (%)	4 (1.1%)	15 (9.6%)	< 0.001
Facilities			
TWMU	69	35	< 0.001
Chiba	135	79	
YCU	147	42	

Abbreviations: Chiba, Chiba University Hospital; PHLF, posthepatectomy liver failure; TWMU, Tokyo Women's Medical University Hospital; YCU, Yokohama City University Hospital.

### 3.1 Comparing Machine Learning Models

The results of the LR, NN, and RF analyses are listed in Table 2.

**TABLE 2:** The results of the LR, NN, and RF analyses for PHLF.

	With ICG					Without ICG				
	LR			NN	RF	LR			NN	RF
	L1	L2	Elastic net			L1	L2	Elastic net		
acc	0.69	0.694	0.691	0.662	0.704	0.7	0.699	0.701	0.692	0.724
auc	0.686	0.69	0.687	0.648	0.723	0.728	0.73	0.728	0.693	0.726
f1	0.154	0.179	0.162	0.295	0.367	0.278	0.284	0.283	0.37	0.394
PPV	0.521	0.522	0.524	0.485	0.529	0.531	0.527	0.536	0.501	0.606
NPV	0.707	0.711	0.708	0.73	0.74	0.724	0.724	0.724	0.751	0.748

Abbreviations: LR, logistic regression; NN, neural network; NPV, negative prediction value; PHLF, posthepatectomy liver failure; PPV, positive prediction value; RF, random forest.

For the ICG‐used model, ACC values were 0.691, 0.662, and 0.704 for the LR, NN, and RF analyses, respectively. The AUCs of LR, NN, and RF analyses were 0.687, 0.648, and 0.723, respectively. The F1 scores for the LR, NN, and RF analyses were 0.162, 0.295, and 0.367, respectively.

**FIGURE 1:**
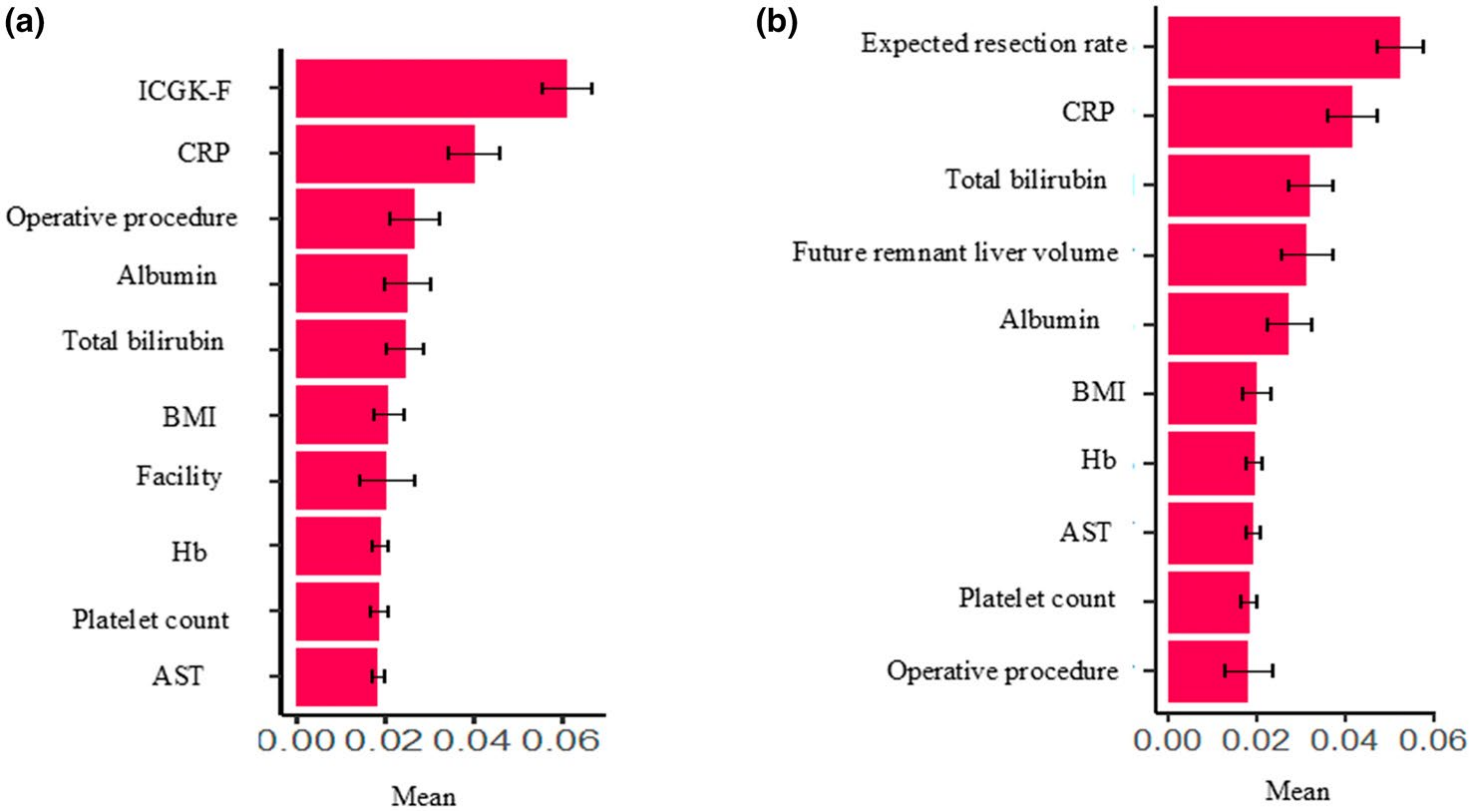
(a) Feature importance of random forest model with ICG predicting posthepatectomy liver failure (PHLF) assessed by SHapley Additive exPlanations. Indocyanine green plasma clearance rate (ICGK) multiplied by the percentage of remaining liver measured by CT volumetry (ICGK‐F), C‐reactive protein (CRP), and operative procedure were extracted as the three highest factors. (b) Feature importance of random forest model without ICG predicting posthepatectomy liver failure (PHLF) assessed by SHapley Additive exPlanations. The predicted resection rates of the liver, C‐reactive protein (CRP), and total bilirubin were extracted as the three highest factors.

For the ICG‐unused model, the ACC values were 0.701, 0.692, and 0.724 for the LR, NN, and RF analyses, respectively. The AUC for LR, NN, and RF analyses were 0.728, 0.693, and 0.726, respectively. The F1 scores for the LR, NN, and RF analyses were 0.283, 0.370, and 0.394, respectively. RF analysis showed the highest ACC, positive prediction value (PPV) and negative prediction value (NPV) in both the ICG‐used and ICG‐unused models. When feature importance was calculated using SHAP analysis, ICGK‐F, CRP, and operative procedure were extracted as the three highest factors in the ICG‐used model, and the predicted resection rates of the liver, CRP, and total bilirubin were extracted in the ICG‐unused model (Figure 1a,b).

### 3.2 Creation of Decision Tree Model

**FIGURE 2:**
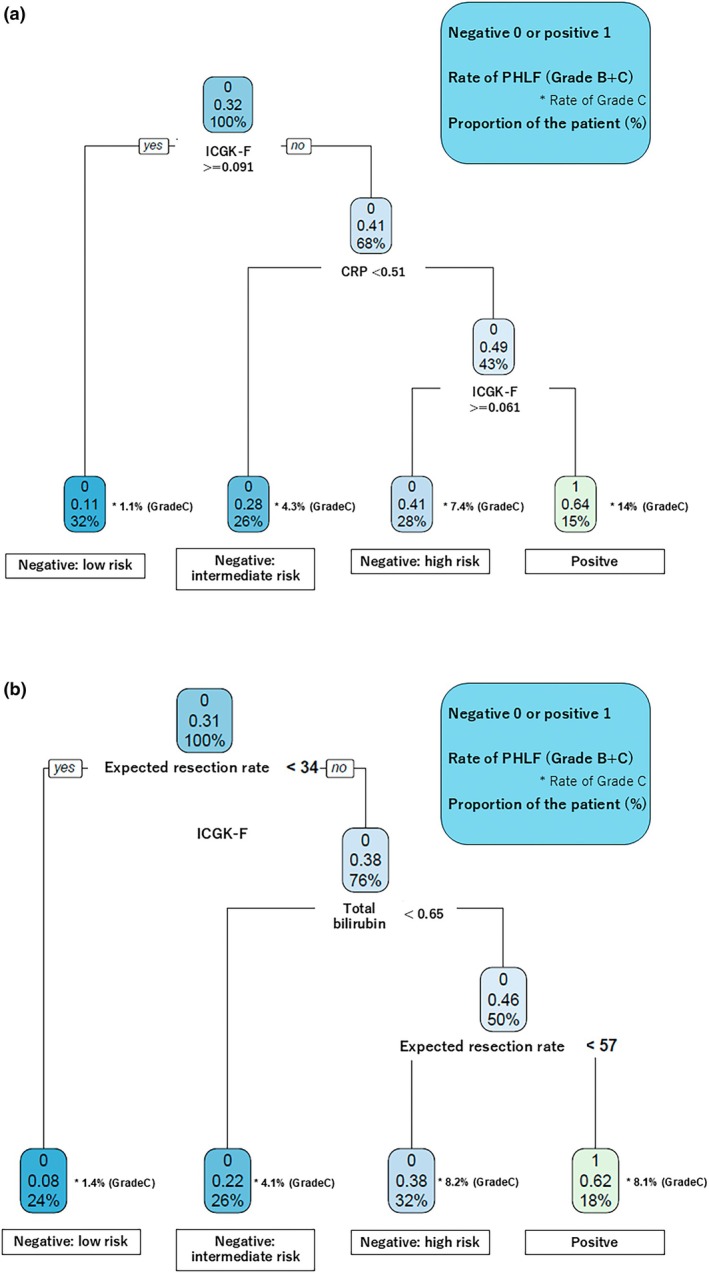
(a) Decision tree model for posthepatectomy liver failure (PHLF) (ICG‐used model). Indocyanine green plasma clearance rate (ICGK) multiplied by the percentage of remaining liver measured by CT volumetry (ICGK‐F) and C‐reactive protein (CRP) were used to create in the ICG‐used model. (b) Decision tree model for posthepatectomy liver failure (PHLF) (ICG‐unused model). The expected liver resection rate and total bilirubin level were used in the ICG‐unused model.

The DT models for the ICG‐used and ICG‐unused samples are shown in Figure 2a,b, respectively.

To improve clinical applicability, we retrained the DT model using the two factors that were among the top three most important variables. ICGK‐F and CRP levels were used in the ICG‐used model, and the expected liver resection rate and total bilirubin level were used in the ICG‐unused model. All continuous variables were automatically evaluated over all potential cutoff points, and the threshold that best separated the outcome variables was selected. Variables for the final decision tree models were selected from the top‐ranked features identified by SHAP analysis, with priority given to clinical interpretability and simplicity rather than maximal complexity.

**FIGURE 3:**
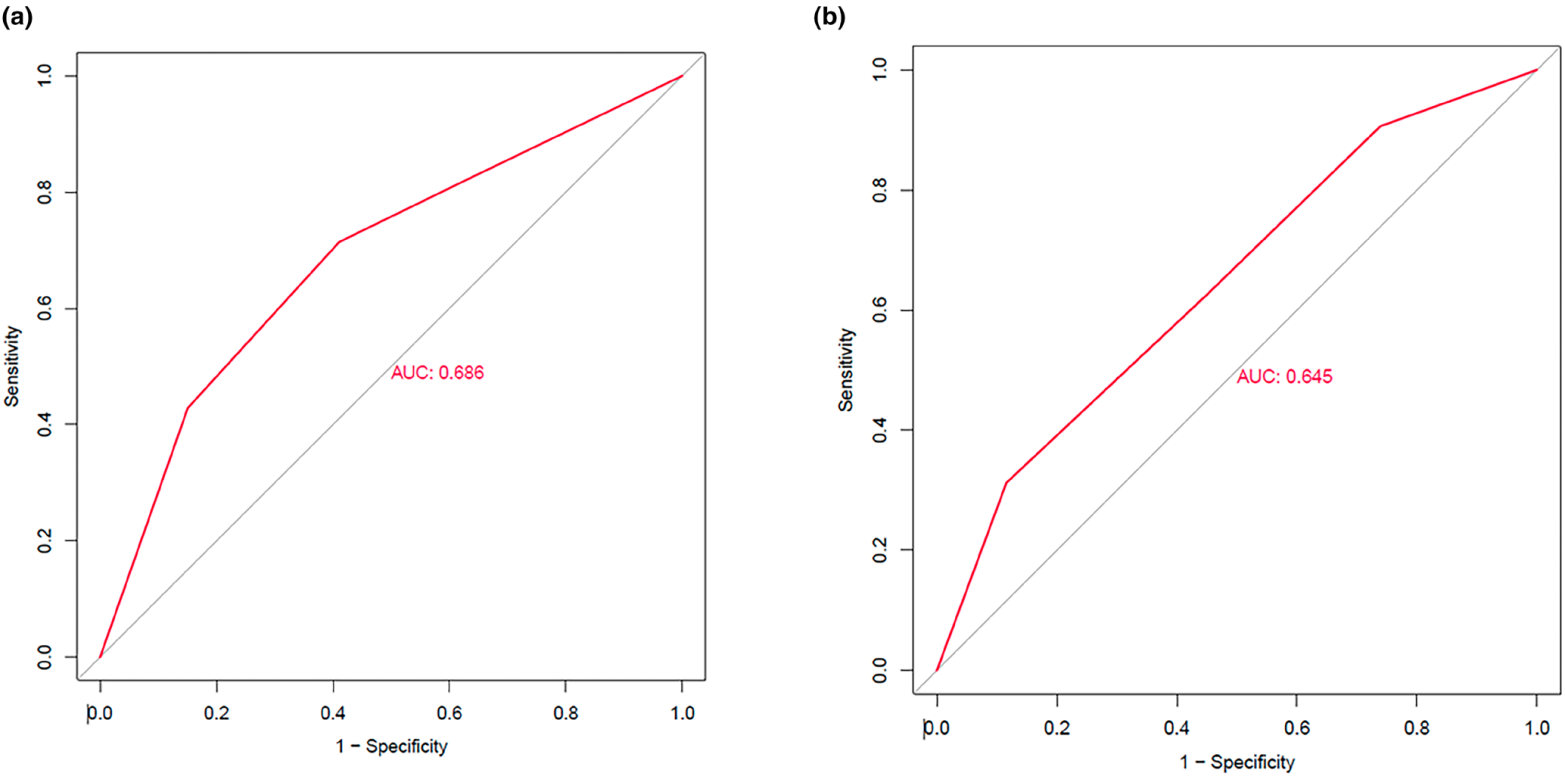
(a) Area under curve of decision tree model (ICG‐used model). (b) Area under curve of decision tree model (ICG‐unused model).

In the constructed DT model, we categorized the cases into negative and positive cases, and negative cases were further classified into three groups depending on their risks: low, intermediate, and high. The proportion of cases diagnosed as PHLF positive was 15% in the ICG‐used model and 18% in the ICG‐unused model. In the positive group, the incidence of PHLF (Grades B and C) was 64% and 62% in the ICG‐used and ICG‐unused models, respectively, and the incidence of Grade C PHLF was 14% and 8.1% in these models, respectively. On using the ICG‐used model in negative cases, the incidence of PHLF (Grades B and C) was 11% in the Negative Low group, 28% in the Negative Intermediate group, and 41% in the Negative High group. The DT also included the incidence of Grade C PHLF, which was 1.1%, 4.3%, and 7.4% in the Negative Low, Intermediate, and High groups, respectively. On using the ICG‐unused model in negative cases, the incidence of PHLF was 8% in the Negative Low group, 22% in the Negative Intermediate group, and 38% in the Negative High group. The DT also included the incidence of Grade C PHLF, which was 1.4%, 4.1%, and 8.2% in the Negative Low, Intermediate, and High groups, respectively. The AUCs of the DT models were 0.686 and 0.645 for the ICG‐used and ICG‐unused models, respectively (Figure 3a,b). Patients classified as positive by the decision tree models also showed higher short‐term mortality rates compared with negative classifications. Furthermore, we examined the conventional liver resection criteria—the Makuuchi criteria and ICGKF value (cutoff 0.05)—in the same cohort and compared their PPV and NPV with our DT model (Table 3).

**TABLE 3:** Positive and negative predictive values and short‐term mortality according to decision tree models with and without indocyanine green.

	DT with ICG		DT without ICG		Makuuchi criteria		ICGK‐*F* < 0.05	
	Positive	Negative	Positive	Negative	Positive	Negative	Positive	Negative
PHLF(Grades B and C) Positive/Negative	52/29	105/321	54/33	104/316	94/151	62/200	26/22	130/329
PPV	0.64		0.62		0.384		0.54	
NPV	0.75		0.754		0.763		0.72	
30‐day mortality	4/82 (4.9%)	6/425 (1.4%)	2/88 (2.3%)	8/419 (1.9%)	6/245 (2.4%)	4/262 (1.5%)	6/48 (12.5%)	4/459 (0.9%)
90‐day mortality	8/82 (9.8%)	11/425 (2.5%)	3/88 (3.4%)	16/419 (3.8%)	12/245 (4.8%)	7/262 (2.7%)	8/48 (16.6%)	11/459 (2.4%)

Abbreviations: DT, Decision Tree; NPV, negative prediction value; PHLF, posthepatectomy liver failure; PPV, positive prediction value.

## 4 Discussion

This study employed ML to develop two simplified and user‐friendly PHLF risk models: an ICG‐used model and an ICG‐unused model. Furthermore, by creating DTs, we were able to construct multi‐layered risk classifications using preoperative test data.

Several models have been developed to predict PHLF [5, 8, 11, 14]. Measuring the FLV during liver resection has become common. However, the function of the remnant liver is more important [15]. Various methods are available to assess future remnant liver function. ICG tests are commonly used in east Asia, especially in Japan. However, ICG clearance rate in patients with prolonged jaundice or constitutional ICG excretory defects often results in an underestimation of the actual liver function [16, 17]. Therefore, the ICG data used in this study represent the final preoperative liver functional status at the time of surgical decision‐making. Accordingly, the present analysis focuses on the physiological condition immediately before hepatectomy, rather than baseline parameters prior to PVE.

In Europe and the United States, ICG tests are rarely performed, and only scintigraphy or Limax tests are performed in a few cases as liver function tests [10, 18]. In addition, indocyanine green is a single diagnostic agent with a relatively limited global market, raising concerns regarding potential supply instability. To ensure robust and sustainable preoperative risk assessment regardless of ICG availability, we developed an alternative ICG‐independent model using routinely available clinical parameters. Therefore, we created two models from the same dataset, one using ICG data and one not using ICG data.

In both the ICG‐used and ICG‐unused models, the unique feature of this study was that each of the three negative groups (Negative Low, Intermediate, and High Risk) was assigned to a specific risk category. Using this model, patients with PHLF‐positive results must wait for FLV escalation via portal vein embolization and undergo reexamination. In the high‐risk group, a similar strategy is employed, and if an increase in FLV is achieved, the patient will transition to an intermediate‐ or low‐risk category, making it possible to perform surgery more safely. Furthermore, safer surgery can be achieved through preoperative management, such as improving cholangitis, lowering CRP and total bilirubin. Increased operative time and blood loss have also been reported as risk factors for PHLF [5, 19]. Surgeons should be more careful in reducing operative time and blood loss, especially in high‐risk patients.

In this study, we performed analyses using LR, NN, and RF, but employed DT for model construction. The reason for this is that models like NN and RF are difficult for clinicians to understand intuitively, making them appear as black boxes internally. By using RF to derive more significant features and creating a DT with fewer variables and fewer layers, we constructed a model that surgeons can easily understand and use. In constructing the decision tree models, variables were selected not only based on their importance ranking but also with consideration of clinical interpretability and simplicity. Although operative procedure and C‐reactive protein were highly ranked in the feature importance analysis, their inclusion resulted in overly complex tree structures without meaningful improvement in discrimination. Because operative procedure reflects surgical strategy rather than intrinsic liver functional reserve, it was intentionally excluded from the final models in the ICG‐used model. In the ICG‐unused model, total bilirubin was selected instead of C‐reactive protein to construct a simpler liver function‐focused model, whereas C‐reactive protein was retained in the ICG‐used model as a complementary predictor.

Although chemotherapy has advanced in recent years, surgical treatment remains the only potentially curative treatment. By showing safety criteria for liver resection based on preoperative data in our DTs, we can avoid missing surgical opportunities due to the previous single conventional criteria.

The AUC for predicting the occurrence of Grades B and C PHLF was similar in both the ICG‐used and ICG‐unused models. The condition of patients with Grade B liver failure improves with noninvasive treatment, and it is not a life‐threatening problem in procedures with a high risk of complications. In this study, we also included Grade C PHLF incidences, which will be helpful in deciding whether resection should be performed.

One limitation of this study is the lack of external validation. Because perihilar cholangiocarcinoma requiring major hepatectomy with extrahepatic bile duct resection is a relatively rare disease, all available cases were used to develop clinically interpretable decision tree models. At this exploratory stage, our primary aim was to establish simple and user‐friendly risk stratification tools rather than definitive predictive models. External and prospective validation in independent cohorts is warranted and will be an important focus of future studies. However, we were able to construct two versatile PHLF risk models, depending on whether ICG was used.

In conclusion, these models may offer a simple and practical approach for predicting the risk of posthepatectomy liver failure and hold promise for future clinical application.

## Author Contributions

**Yuki Homma:** conceptualization, investigation, writing – original draft, writing – review and editing, visualization. **Hiroki Ohya:** conceptualization, methodology, writing – review and editing. **Ryusei Matsuyama:** investigation, writing – review and editing. **Isamu Hosokawa:** conceptualization, investigation, writing – review and editing. **Tsukasa Takayashiki:** conceptualization, investigation, writing – review and editing. **Yusuke Ome:** conceptualization, investigation, writing – review and editing. **Shuichiro Uemura:** conceptualization, investigation, writing – review and editing. **Ryota Higuchi:** conceptualization, investigation, writing – review and editing. **Goro Honda:** writing – review and editing, supervision, conceptualization. **Eiryo Kawakami:** conceptualization, writing – original draft, writing – review and editing, methodology, data curation, formal analysis, visualization. **Masayuki Ohtsuka:** conceptualization, writing – review and editing. **Itaru Endo:** conceptualization, writing – review and editing.

## Funding

The authors have nothing to report.

## Ethics Statement

This study was approved by the Institutional Review Board of Yokohama City University (Approval No. F220100002).

## Consent

The requirement for informed consent was waived by the opt‐out method.

## Conflicts of Interest

Dr. Itaru Endo serves as an Editorial Board Member of *Annals of Gastroenterological Surgery*. The other authors declare no conflicts of interest.
